# Costimulation of Murine Osteoblasts with Interferon-*γ* and Tumor Necrosis Factor-*α* Induces Apoptosis through Downregulation of Bcl-2 and Release of Cytochrome *c* from Mitochondria

**DOI:** 10.1155/2018/3979606

**Published:** 2018-08-09

**Authors:** Mayumi Iguchi, Miki Hiroi, Haruhide Kanegae, Yoshihiro Ohmori

**Affiliations:** ^1^Division of Orthodontics, Department of Human Development and Fostering, Meikai University School of Dentistry, 1-1 Keyakidai, Sakado, Saitama 350-0283, Japan; ^2^Division of Microbiology and Immunology, Department of Oral Biology and Tissue Engineering, Meikai University School of Dentistry, 1-1 Keyakidai, Sakado, Saitama 350-0283, Japan

## Abstract

During chronic inflammation from diseases, such as periodontal disease, the proinflammatory cytokines interferon-gamma (IFN*γ*) and tumor necrosis factor-*α* (TNF*α*) alter bone remodeling. To elucidate the underlying molecular mechanisms, we investigated the effect of IFN*γ* and TNF*α* on the proliferation and survival of clonal MC3T3-E1 mouse osteoblasts. We found that although IFN*γ* or TNF*α* alone affected cell growth and survival only marginally, costimulation with both synergistically inhibited cell growth and reduced cell viability. The diminished cell viability was due to apoptosis, as indicated by increased TUNEL staining and elevated caspase 3, 8, and 9 activities. Western blot also showed that costimulation with IFN*γ* and TNF*α* elicited cytochrome *c* release and downregulated B cell lymphoma 2 (Bcl-2) expression without affecting Bcl-2-associated X (Bax) protein expression. Furthermore, stable Bcl-2 overexpression significantly alleviated cell death following costimulation. Collectively, these results suggested that IFN*γ* and TNF*α* elicited osteoblast apoptosis via cytochrome *c* release from damaged mitochondria, caspase activation, and Bcl-2 downregulation.

## 1. Introduction

Physiological bone remodeling is achieved via balance between bone resorption by osteoclasts and bone formation by osteoblasts [1–4]. Accordingly, bone degradation, which is often seen in periodontal disease, occurs when inflammatory cytokines in the bone microenvironment shift this balance by activating osteoclasts but suppressing osteoblast proliferation/differentiation and inducing osteoblast apoptosis [5–8]. In particular, tumor necrosis factor-*α* (TNF*α*) and interferon-gamma (IFN*γ*) are believed to promote bone degradation in periodontal disease, because periodontal tissues in patients with periodontitis are infiltrated by CD4^+^ and CD8^+^ T cells, which produce these cytokines [9–14].

TNF*α* is primarily produced by macrophages and T cells but exerts diverse effects on many cell types [15, 16]. For example, TNF*α* inhibits bone formation via multiple mechanisms, including inhibition of osteoblast differentiation and mineralization and suppression of type I collagen synthesis and alkaline phosphatase activity [14, 17–19]. TNF*α* also differentiates precursor cells into osteoclasts and promotes inflammatory bone resorption [20]. On the other hand, IFN*γ* is produced by T-helper 1 (Th1) cells to promote cell-mediated immunity [21, 22]. Additionally, IFN*γ* suppresses alkaline phosphatase activity in osteoblasts, downregulates bone *gla* protein [23, 24], and promotes differentiation of mesenchymal stem cells into osteoblasts [25]. Unlike TNF*α* and interleukin-1, IFN*γ* directly suppresses osteoclast differentiation by interfering with receptor activator of nuclear factor-*κ*B ligand (RANKL) signaling [26, 27] but indirectly stimulates osteoclast formation and promotes bone resorption through production of RANKL and TNF*α* by stimulating T cell activation [28].

Although TNF*α* and IFN*γ* individually affect osteoblast activity and viability only marginally [19, 29], costimulation with both induces production of nitric oxide (NO), which inhibits osteoblast differentiation and promotes apoptosis [29–35]. However, the molecular mechanisms driving these events are not fully understood. Therefore, in this study, we investigated cytokine-induced cell death in mouse MC3T3-E1 osteoblasts, finding that costimulation with TNF*α* and IFN*γ* induced cytochrome *c* release from mitochondria, activated caspases, and downregulated B cell lymphoma 2 (Bcl-2) expression.

## 2. Materials and Methods

### 2.1. Reagents

Recombinant mouse IFN*γ* and mouse TNF*α* were obtained from Chemicon International (Temecula, CA, USA) and R&D Systems (Minneapolis, MN, USA), respectively. Puromycin, digitonin, and protease inhibitor cocktail were purchased from Sigma-Aldrich (St. Louis, MO, USA).

### 2.2. Cell Culture

MC3T3-E1 cells, which are osteoblasts derived from mouse calvaria [36–38], were seeded in 10 cm plastic cell culture plates (Becton Dickinson, Franklin Lakes, NJ, USA) at 1.5 × 10^5^ cells per plate in *α*-minimum essential medium (*α*-MEM; Invitrogen, Grand Island, NY, USA) containing 10% inactivated fetal bovine serum (FBS; Bio West, Miami, FL, USA) and 1% penicillin G-streptomycin sulfate (Invitrogen). Cells were incubated at 37°C in 5% CO_2_ and passaged every 3 days. To passage, the cells were first washed with phosphate-buffered saline (PBS) and then with PBS containing 0.02% EDTA, disaggregated into single cells using 0.1% actinase E (Kaken Pharmaceutical, Tokyo, Japan), and diluted to the desired density.

### 2.3. Cell Proliferation

Cells were seeded on 3 cm culture plates (Becton Dickinson) at 5 × 10^4^ cells per plate and cultured for 24 h at 37°C and 5% CO_2_ in *α*-MEM with 10% FBS, followed by treatment with IFN*γ* and TNF*α* for various durations. The cells were then detached from cell culture plates using 0.1% actinase E and counted on a hemocytometer (Erma, Tokyo, Japan).

### 2.4. Cell Viability

Cell viability was measured using a cell counting kit (Dojindo, Kumamoto, Japan) based on the formation of water-soluble formazan from the tetrazolium salt WST-8 (2-[2-methoxy-4-nitrophenyl]-3-[4-nitrophenyl]-5-[2,4-disulfophenyl]-2*H*-tetrazolium, monosodium salt) [39]. Briefly, cells were seeded at 1.5 × 10^3^ cells per well in a 96-well microplate (Becton Dickinson), cultured for 24 h at 37°C and 5% CO_2_ in *α*-MEM with 1% FBS, and treated with IFN*γ* and TNF*α* for various durations. The cells were then stained with 10 *μ*l WST-8 reagent for 2 h at 37°C and 5% CO_2_ and assayed at 450 nm on a microplate reader (Multiskan Bichromatic; Labsystems Diagnostics, Helsinki, Finland). Cell viability was normalized to that of unstimulated cells, which was set at 100%. Cytotoxicity was then calculated as the ratio of cell viability of unstimulated cells to that of cytokine-treated cells. Cell viability and cytotoxicity were also measured in the same manner in MC3T3-E1 cells precultured for 5 days to form a confluent monolayer.

### 2.5. DNA Fragmentation by Terminal Deoxynucleotidyl Transferase dUTP Nick-End Labeling (TUNEL) Assay

The Apo Alert DNA fragmentation assay kit (BD Biosciences, San Jose, CA, USA) was used for TUNEL assays [40]. Briefly, cells were seeded in each well of a Lab-Tek chamber slide (Nalge Nunc International, Rochester, NY, USA) at 6 × 10^4^ cells per well, cultured for 72 h to form a monolayer, and treated with IFN*γ* and TNF*α* for various durations. The cells were subsequently washed with PBS, fixed using 5% acetic acid in ethanol, washed again with PBS, and treated with 0.2% Triton X-100 for 5 min. After washing, the cells were equilibrated for 10 min at room temperature in 50 *μ*l equilibration buffer supplied with the kit, followed by staining at 37°C for 1 h with 45 *μ*l equilibration buffer, 5 *μ*l nucleotide mix, and 1 *μ*l TdT enzyme, which incorporates FITC-labeled dUTPs onto the 3′-terminal hydroxyl group of DNA fragments. Nuclei were then counterstained with propidium iodide (PI), and TUNEL-stained (apoptotic) cells were enumerated using a confocal laser microscope (Carl Zeiss, Oberkochen, Germany) at an excitation wavelength of 494 nm and an emission wavelength of 518 nm. The number of apoptotic cells was normalized to the total number of PI-stained cells.

### 2.6. Caspase Activity

Caspase activity was measured using an APOPCYTO caspase colorimetric assay kit (Medical & Biological Laboratories, Nagoya, Japan). Briefly, cells were seeded on 10 cm tissue culture plates at 5 × 10^5^ cells per plate, cultured for 72 h at 37°C and 5% CO_2_ in *α*-MEM with 10% FBS to form a monolayer, and exposed to IFN*γ* and TNF*α* for various durations. The cells were subsequently washed with PBS, harvested with a cell scraper (Costar, Corning, NY, USA), transferred to a 1.5 ml centrifuge tube on ice, and centrifuged for 3 min at 1700*g* and 4°C. The resulting pellet was resuspended in 100 *μ*l cell lysis buffer, gently mixed, placed on ice for 10 min, and centrifuged for 5 min at 9000*g* and 4°C to obtain cell extracts. The extracts were assayed for total protein by the Bradford method [41] using a commercially available reagent (Bio-Rad, Hercules, CA, USA). After diluting extracts to a uniform concentration of protein, caspases were assayed for 1 h at 37°C using DEVD-pNA, IETD-pNA, and LEHD-pNA, which are cleaved by caspase 3, caspase 8, and caspase 9, respectively. The amount of liberated *p*-nitroaniline, a colorimetric marker, was measured by absorbance at 405 nm on a microplate reader (Multiskan Bichromatic; Labsystems Diagnostics). Caspase activity was quantified based on a *p*-nitroaniline standard curve. The enzymatic activity in unstimulated cells was set to 1, to which the enzymatic activity in cytokine-stimulated cells was normalized.

### 2.7. Mitochondrial Fractionation and Cytochrome *c* Release

Cytochrome *c* release was evaluated according to published methods, with some modifications [42, 43]. The cells were cultured as described for the caspase assay, washed three times using ice-cold PBS, washed once with PBS containing 0.02% EDTA, and disaggregated into single cells using 0.1% actinase E. The cells were then pelleted by centrifugation for 3 min at 1700*g* and 4°C; gently suspended in permeabilization buffer (220 mM mannitol, 68 mM sucrose, 80 mM KCl, 10 mM HEPES (pH 7.4), 2.5 mM EGTA, 1 mM EDTA, 1 mM DTT, 200 *μ*g/ml digitonin, and 1% protease inhibitor cocktail); placed on ice for 5 min; and centrifuged for 5 min at 750*g* and 4°C. The resulting supernatant was used as the cytoplasmic fraction, while the pellet was resuspended in 50 *μ*l cell lysis buffer (50 mM Tris-HCl (pH 7.4), 150 mM NaCl, 2 mM EGTA, 2 mM EDTA, 0.2% Triton X-100, 0.3% NP-40, and 1 mM DTT) and centrifuged for 10 min at 8500*g* and 4°C to pellet nuclei. The resulting supernatant was used as the mitochondrial fraction. Proteins in both fractions were quantified by the Bradford method [41] and adjusted to a uniform protein concentration using sodium dodecyl sulfate (SDS) sample buffer (250 mM Tris-HCl (pH 6.8), 8% SDS, 20% 2-mercaptoethanol, 40% glycerol, and 0.04% bromophenol blue).

### 2.8. Western Blot

Protein samples obtained as described were subjected to 12% SDS-polyacrylamide gel electrophoresis (PAGE) and transferred to a polyvinylidene difluoride membrane (Millipore, Bedford, MA, USA) using a semidry transfer cell (Bio-Rad). Membranes were then blocked for 1 h at room temperature with Tris-buffered saline-Tween 20 (TBS-T; 20 mM Tris-HCl (pH 7.4), 137.5 mM NaCl, and 0.1% Tween 20) containing 5% skim milk, washed with TBS-T three times, and probed at 4°C for 12 h with rabbit antibodies to sheep cytochrome *c* (Sigma-Aldrich), human Bcl-2 (1 : 500 dilution; Santa Cruz Biotechnology, Dallas, TX, USA), and human Bcl-2-associated X protein (Bax; 1 : 500 dilution; Santa Cruz Biotechnology) or with goat antibodies to human *β*-actin (1 : 500 dilution; Santa Cruz Biotechnology). Subsequently, the membranes were washed three times with TBS-T, labeled for 1 h at room temperature with appropriate secondary antibodies conjugated to horseradish peroxidase (1 : 2000 dilution; Amersham Biosciences, Piscataway, NJ, USA), washed, stained with West Pico chemiluminescent substrate (Pierce, Rockford, IL, USA), and visualized on X-ray film (KODAK BioMax XAR Film; KODAK, Rochester, NY, USA).

### 2.9. Establishment of Cells Stably Expressing Bcl-2

Cells stably expressing Bcl-2 were established by transforming MC3T3-E1 cells with a Bcl-2 expression vector (pCMV-Bcl-2) and a plasmid with a puromycin-resistance gene (pBabePuro). The former [44] was provided by Dr. Shie-Liang Hsieh (Institute and Department of Microbiology and Immunology, National Yang-Ming University, Taipei, Taiwan), whereas the latter was provided by Dr. Charles S. Tannenbaum (Department of Immunology, Cleveland Clinic Foundation, Cleveland, OH, USA). Briefly, the cells were seeded on 10 cm tissue culture plates at 3 × 10^5^ cells per plate in *α*-MEM containing 10% FBS, cultured for 24 h at 37°C and 5% CO_2_, and transfected with 0.1 *μ*g pBabePuro in PolyFect transfection reagent (Qiagen, Hilden, Germany) along with either 8 *μ*g pCMV-Bcl-2 or 8 *μ*g control vector (pcDNA3; Invitrogen). After 24 h, 2 *μ*g/ml puromycin was added to the media, which were refreshed every 3 days. Antibiotic-resistant colonies were grown for 2 weeks, isolated using cloning rings (Iwaki, Tokyo, Japan), detached from the tissue culture plate using 0.1% actinase E, and repassaged in 24-well microplates. Twenty colonies each of the cells transfected with pCMV-Bcl-2 or pcDNA3 were harvested, and Bcl-2 protein expression was confirmed by western blot.

### 2.10. Statistical Analysis

Student's *t*-test for paired data was used to test for statistically significant differences using Prism 5 software (GraphPad Software, La Jolla, CA, USA). One-way analysis of variance (ANOVA) was used to compare multiple groups. A *p* < 0.05 was considered statistically significant.

## 3. Results

### 3.1. Costimulation with IFN*γ* and TNF*α* Suppresses Proliferation of MC3T3-E1 Mouse Osteoblasts

**Figure 1:**
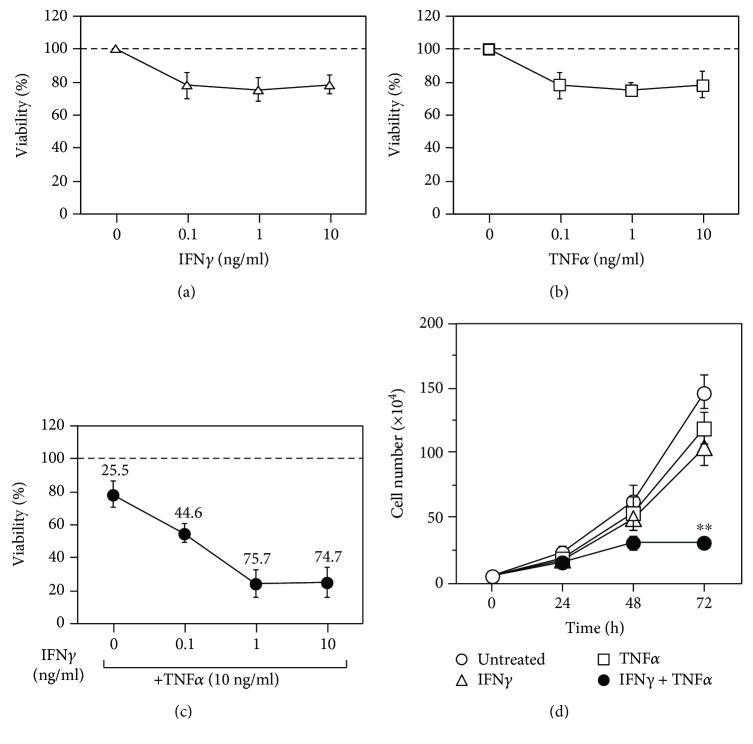
Effects of IFN*γ* and TNF*α* on the growth of MC3T3-E1 mouse osteoblasts. Exponentially growing MC3T3-E1 mouse osteoblasts were seeded in 96-well plates, incubated for 24 h, and treated for 72 h with various concentrations of IFN*γ* (a), TNF*α* (b), or both (c). Cell viability was determined, and the percentage of cytotoxicity is shown above the symbols. Data represent the mean ± SEM of three independent experiments. The exponentially growing MC3T3-E1 mouse osteoblasts were also seeded in 3 cm dishes, incubated for 24 h, stimulated with or without 10 ng/ml IFN*γ* and/or 5 ng/ml TNF*α*, and analyzed at various time points (d), with time 0 indicating the addition of cytokines. Viable cells were counted using a hemocytometer. Data represent the mean ± SEM of three independent experiments. ^∗∗^*p* < 0.01 versus untreated cultures by Student's *t*-test.

IFN*γ* (Figure 1(a)) and TNF*α* (Figure 1(b)) alone marginally decreased cell proliferation at 0.1 ng/ml, with viability decreasing by only ~20%, even at 10 ng/ml. However, stimulation with varying concentrations of IFN*γ* in the presence of 10 ng/ml TNF*α* significantly decreased cell viability to ~20% at both 1 ng/ml and 10 ng/mL IFN*γ* (Figure 1(c)). Similar results were obtained at 5 ng/ml TNF*α* (data not shown). These results indicated that stimulation with either IFN*γ* or TNF*α* did not strongly affect MC3T3-E1 cell proliferation, but costimulation with both led to synergistic suppression of cell growth.

Time course analysis following exposure to IFN*γ* and TNF*α* (Figure 1(d)) indicated slight suppression of the growth of MC3T3-E1 cells stimulated with either IFN*γ* or TNF*α* alone, although cells continued to proliferate, even after 72 h. By contrast, costimulation with both cytokines led to marked suppression of cell growth by 72 h.

### 3.2. Effects of IFN*γ* and TNF*α* Costimulation on Confluent MC3T3-E1 Cells

**Figure 2:**
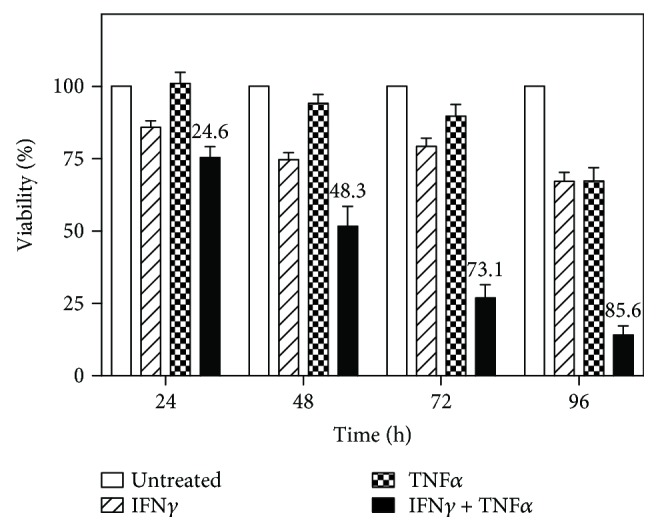
IFN*γ* and TNF*α* costimulation is cytotoxic to confluent MC3T3-E1 monolayers. Cells were seeded in 96-well plates, incubated for 5 days to form a confluent monolayer, and treated with 10 ng/ml IFN*γ* and/or 5 ng/ml TNF*α*. Cell viability was monitored over time. Data represent the mean ± SEM of three independent experiments, with the percentage of cytotoxicity indicated.

With fresh media provided every 3 days to prevent nutrient depletion, MC3T3-E1 cells were grown for 5 days to form a confluent monolayer, which exhibited osteoblastic properties [37, 45, 46]. As measured by WST-8 assay (Figure 2), stimulation of these monolayers with either IFN*γ* or TNF*α* alone did not strongly affect cell viability, whereas costimulation with both led to a time-dependent loss of cell viability to between ~20% and ~25% within 72 h. These results indicated that the costimulation with IFN*γ* and TNF*α* was cytotoxic to MC3T3-E1 monolayers.

### 3.3. Costimulation with IFN*γ* and TNF*α* Causes DNA Fragmentation in MC3T3-E1 Cells

**Figure 3:**
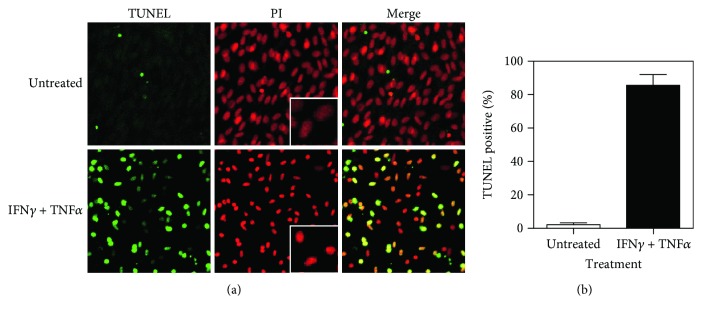
Induction of apoptosis in MC3T3-E1 cells by costimulation with IFN*γ* and TNF*α*. (a) Cells were seeded in Lab-Tek chamber slides, incubated for 3 days to form a confluent monolayer, and treated with 10 ng/ml IFN*γ* and 5 ng/ml TNF*α* for 48 h. Cells were then TUNEL stained to detect DNA fragmentation and stained with PI to detect nuclear condensation. Representative confocal laser-scanning micrographs from three separate experiments are shown. Original magnification, 40x. (b) TUNEL-stained cells accumulated in cultures stimulated with IFN*γ* and TNF*α*. Data represent the mean ± SEM of three independent experiments.

To investigate the mechanisms associated with cytotoxicity, confluent MC3T3-E1 monolayers were stimulated with IFN*γ* and TNF*α* for 72 h, fixed, and TUNEL stained to detect DNA fragmentation, a hallmark of apoptosis, along with PI staining of nuclei and imaging with a confocal laser microscope (Figure 3(a)). The costimulation with IFN*γ* and TNF*α* increased the proportion of TUNEL-stained cells to 80% at 72 h (Figure 3(b)). Additionally, nuclei were more condensed in cells treated with both cytokines than in unstimulated cells (Figure 3(a), middle panel). These results suggested that IFN*γ*- and TNF*α*-induced cytotoxicity was a consequence of apoptosis.

### 3.4. Costimulation with IFN*γ* and TNF*α* Increases Caspase Activity in MC3T3-E1 Cells

**Figure 4:**
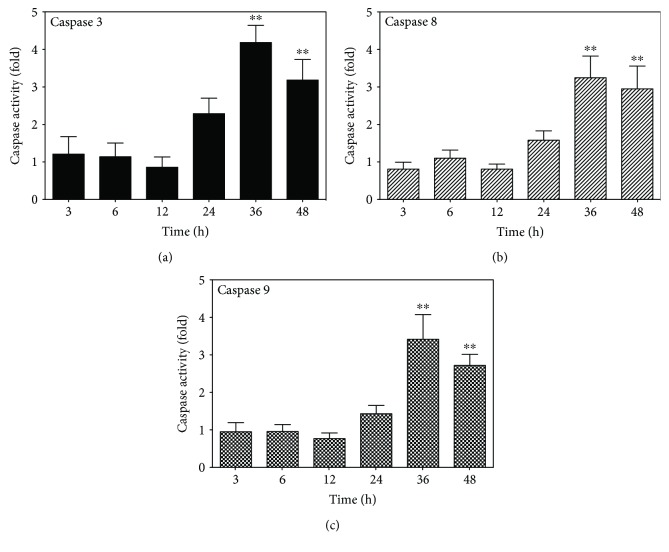
Time-dependent activation of caspases in MC3T3-E1 cells costimulated with IFN*γ* and TNF*α*. Cells were seeded in 10 cm dishes, incubated for 5 days to form a confluent monolayer, and costimulated with 10 ng/ml IFN*γ* and 5 ng/ml TNF*α*. Caspase activity in a 50 *μ*g total cell lysate was monitored over time against appropriate synthetic chromogenic substrates. Relative caspase activity was calculated and compared with caspase activity in untreated cells. Data represent the mean ± SEM of five independent experiments. ^∗∗^*p* < 0.01 versus the 3 h time point by one-way ANOVA.

Apoptosis is induced following activation of cysteine-aspartic proteases (caspases), which cleave various intracellular molecules, including caspase-activated DNase, which fragments chromosomal DNA [47, 48]. Therefore, we investigated whether caspases mediated the apoptotic effects observed following IFN*γ* and TNF*α* costimulation. Caspase 3, 8, and 9 activities were measured over time in confluent MC3T3-E1 cells against peptide substrates labeled with a chromogen (Figures 4(a)–4(c)). We observed no caspase activation within the first 12 h after costimulation with IFN*γ* and TNF*α*; however, caspases 3, 8, and 9 were activated after 24 h, with activity significantly increasing after 36 h (*p* < 0.01). These results implied that the costimulation of MC3T3-E1 cells with IFN*γ* and TNF*α* induced apoptosis via caspase activity.

### 3.5. Costimulation with IFN*γ* and TNF*α* Increases Cytochrome *c* Release and Suppression of Bcl-2 Expression in MC3T3-E1 Cells

**Figure 5:**
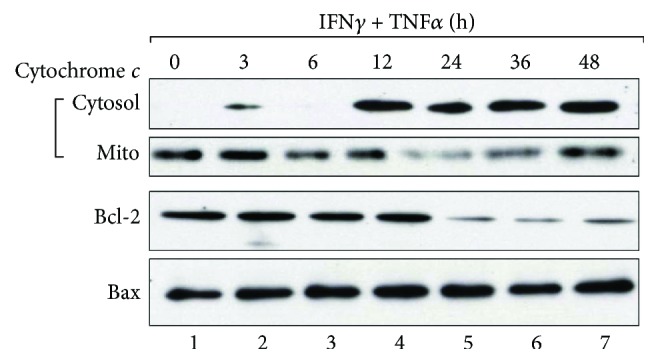
Effect of IFN*γ* and TNF*α* costimulation on cytochrome *c*, Bcl-2, and Bax levels in MC3T3-E1 cells. Cells were cultured as described in Figure 4 and treated with 10 ng/ml IFN*γ* and 5 ng/ml TNF*α*. Samples were collected at various time points and fractionated into cytosolic and mitochondrial (Mito) fractions, of which 20 *μ*g protein was subjected to 12% SDS-PAGE and immunoblotted with antibodies to cytochrome *c*. The mitochondrial fraction was also analyzed by western blot for Bcl-2 and Bax. Results are representative of three independent experiments.

Various apoptotic signals are eventually integrated in mitochondria, which then become permeable and release cytochrome *c* [49–56]. Therefore, cytosolic and mitochondrial fractions from confluent MC3T-E1 monolayers costimulated with IFN*γ* and TNF*α* were separated by SDS-PAGE, and the levels of cytochrome *c* were analyzed by western blot (Figure 5). Cytochrome *c* significantly accumulated in the cytoplasm beginning at 12 h and up to 48 h after costimulation, while cytochrome *c* levels diminished in the mitochondrial fraction. These results suggested that costimulation with IFN*γ* and TNF*α* increased mitochondrial membrane permeability and induced cytochrome *c* release. Additionally, we examined the expression of Bcl-2 and Bax, both of which regulate mitochondrial membrane permeabilization [56–59]. Bcl-2 expression was stable up to 12 h after costimulation with IFN*γ* and TNF*α* but diminished starting at 24 h after stimulation. By contrast, Bax was stable throughout the experiment. These results implied that costimulation with IFN*γ* and TNF*α* shifted the equilibrium between Bcl-2 and Bax in mitochondria, resulting in permeabilization of the mitochondrial membrane.

### 3.6. Increased Bcl-2 Expression in MC3T3-E1 Cells Alleviates Injury due to IFN*γ* and TNF*α*

**Figure 6:**
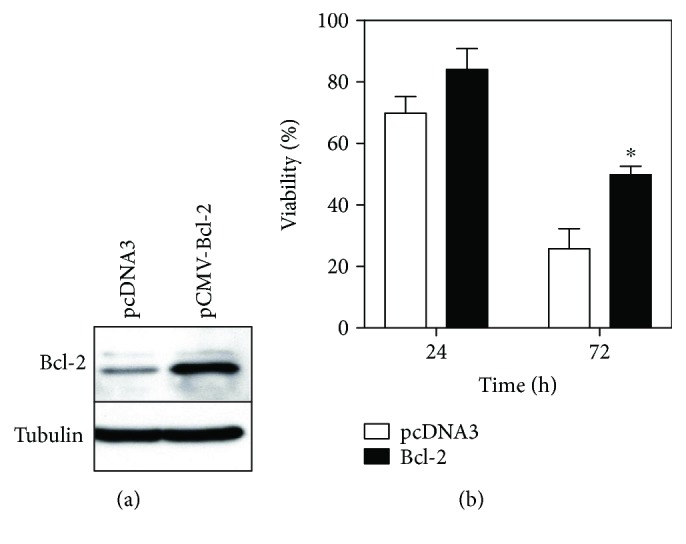
Overexpression of Bcl-2 attenuates IFN*γ*- and TNF*α*-induced cytotoxicity in MC3T3-E1 cells. (a) Western blot for Bcl-2 in cells stably expressing Bcl-2. Total cell lysates were prepared from cells stably transfected with control vector (pcDNA3) or a Bcl-2 expression vector (pCMV-Bcl-2), of which 20 *μ*g protein was subjected to 12% SDS-PAGE and analyzed with antibodies to Bcl-2. The blot was stripped and reprobed with anti-tubulin to confirm loading of equal amounts of total protein. (b) Effect of IFN*γ* and TNF*α* costimulation on the viability of cells stably expressing Bcl-2. Cells stably transfected with control vector or pCMV-Bcl-2 were seeded in 96-well plates, incubated for 5 days to form a confluent monolayer, and costimulated with 10 ng/ml IFN*γ* and 5 ng/ml TNF*α*. Cell viability was monitored over time. Data represent the mean ± SEM of three independent experiments. ^∗^*p* < 0.05 versus control cells by Student's *t*-test.

Because Bcl-2 inhibits mitochondrial membrane permeabilization through Bax [56–59], we investigated whether Bcl-2 overexpression alleviates the apoptotic effects of IFN*γ* and TNF*α*. We transformed MC3T3-E1 cells with either a Bcl-2 expression vector (pCMV-Bcl2) or a control vector (pCDNA3) along with a puromycin-resistance plasmid (pBabePuro), selected drug-resistant colonies, and screened these colonies by western blot for stable Bcl-2 expression (Figure 6(a)). Clones strongly expressing Bcl-2, as well as those transformed with the control vector, were then costimulated with IFN*γ* and TNF*α*. Strikingly, cytotoxicity was significantly alleviated in Bcl-2-expressing clones, as shown in Figure 6(b). These results indicated that apoptosis induced by IFN*γ* and TNF*α* costimulation was partially relieved by Bcl-2-mediated suppression of mitochondrial membrane permeabilization.

## 4. Discussion

Inflammatory bone loss in periodontal disease is due not only to increased bone resorption by osteoclasts but also to suppressed bone formation by osteoblasts [5–8]. In this study, we examined the effect of the inflammatory cytokines IFN*γ* and TNF*α* on the proliferation and viability of MC3T3-E1 mouse osteoblasts and investigated the underlying molecular mechanisms. The data showed that costimulation with IFN*γ* and TNF*α* promoted apoptosis, as indicated by increased DNA fragmentation according to TUNEL staining. Nuclear condensation, another hallmark of apoptotic cells, was also observed based on PI staining. Additionally, IFN*γ* and TNF*α* costimulation activated caspases, which are effectors of apoptosis, and induced cytochrome *c* release from mitochondria. Furthermore, the expression of Bcl-2, a protein that regulates mitochondrial membrane permeability, diminished after the IFN*γ* and TNF*α* costimulation. Collectively, these findings strongly suggested that IFN*γ* and TNF*α* induced apoptosis in MC3T3-E1 cells through mitochondrial damage.

Mitochondria are master regulators of apoptosis and contain apoptosis-inducing proteins, such as cytochrome *c* and Smac/Diablo, between the outer and inner membranes [49–56]. These proteins leak into the cytoplasm when an apoptotic signal permeabilizes the mitochondrial membrane. Subsequently, released cytochrome *c* binds apoptotic protease-activating factor-1 (Apaf-1) in the presence of ATP, thereby inducing apoptosis by activating caspases 9 and 3, initiator and apoptotic caspases, respectively. Additionally, Bcl-2 proteins control mitochondrial membrane permeability and are subdivided into two major functional groups [56–62]. The first group includes Bax and Bcl-2 homologous antagonist/killer (Bak1), which promote mitochondrial membrane permeabilization by inducing structural changes in voltage-dependent anion channels in the mitochondrial outer membrane. By contrast, Bcl-2 sequesters Bax to suppress membrane permeabilization. Accordingly, we investigated the kinetics of Bcl-2 and Bax in MC3T3-E1 cells costimulated with IFN*γ* and TNF*α* (Figure 5). The data showed that Bcl-2 expression was suppressed in the mitochondrial fraction after 24 h of stimulation, although no major changes in Bax expression were observed, implying that the costimulation with IFN*γ* and TNF*α* might alter the equilibrium between Bcl-2 and Bax, liberate Bax, and promote mitochondrial membrane permeabilization. Conversely, Bcl-2 overexpression significantly alleviated cell death induced by IFN*γ* and TNF*α* costimulation (Figure 6). These results suggested that the apoptotic effects of IFN*γ* and TNF*α* were dependent upon Bcl-2/Bax equilibrium.

Interestingly, cytochrome *c* release was observed beginning at 12 h after costimulation, whereas Bcl-2 expression was suppressed at 24 h (Figure 5). Caspase activation was also observed at 24 h, coinciding with decreased Bcl-2 levels. These results suggested that the initiating event after IFN*γ* and TNF*α* costimulation was cytochrome *c* release from damaged mitochondria, followed by caspase activation and Bcl-2 degradation [42, 63]. The initial mitochondrial damage might be due to increased NO concentration, given that IFN*γ* and TNF*α* costimulation induces the expression of NO synthase-2 (Nos2) in osteoblasts [29–33, 35]. Endogenous reactive oxygen species (ROS) might also contribute to mitochondrial damage [64], because IFN*γ* induces the expression of NADPH oxidase-1, which generates ROS, such as superoxide [65]. Indeed, preliminary experiments with the antioxidant *N*-acetyl-L-cysteine partially reduced cell death and caspase activation induced by IFN*γ* and TNF*α* costimulation (data not shown). Taken together, these observations suggest that although IFN*γ* or TNF*α* alone only modestly affected osteoblast viability, the costimulation with both might boost NO and ROS concentrations, thereby causing mitochondrial membrane damage and cytochrome *c* release. Alternatively, the costimulation might suppress the expression of intracellular antioxidants, such as superoxide dismutase and catalase, thereby allowing ROS accumulation and promoting mitochondrial damage. In any case, the mechanisms underlying the observed mitochondrial damage will be the subject of future research.

It is important to confirm that the costimulation with IFN*γ* and TNF*α* leads to induction of apoptosis in other osteoblastic cell lines. Although few studies have described osteoblastic apoptosis induced by TNF*α* and IFN*γ* costimulation in cell lines other than MC3T3-E1, a previous study has shown that stimulation of rat osteoblast cell line ROS 17/28 with a mixture of TNF*α*, IFN*γ*, and LPS induced apoptosis [34]. One reason why limited publications of this nature exist is because human osteoblastic cell lines such as MG-63 and Saos-2 were established from osteosarcoma, resulting in the loss of their normal physiological properties, and many tumor cells have been shown to acquire antiapoptotic pathways. In contrast, MC3T3-E1 cells were established from newborn mouse calvaria and have been shown to possess the features of normal osteoblasts. Further study using human osteoblastic cell lines with normal physiological properties will be necessary to corroborate the osteoblastic apoptosis induced by costimulation with IFN*γ* and TNF*α*.

Although IFN*γ* directly inhibits osteoclastogenesis by interfering with the RANKL-RANK signaling pathway and inducing apoptosis mediated by Fas/Fas ligand signaling [27, 66], accumulating evidence suggests that Th1-derived IFN*γ* and TNF*α* induced by acquired immune response promote net bone loss in pathological conditions such as periodontitis [28, 67–70]. A mouse model of alveolar bone loss induced by periodontopathic bacteria *Porphyromonas gingivalis* showed that CD4^+^ T cell-derived IFN*γ* and IL-6 were important effectors of bone loss associated with periodontal disease [67]. Peripheral blood mononuclear cells (PBMCs) from patients with chronic periodontitis have been shown to produce large amounts of inflammatory cytokines including IFN*γ* and TNF*α* and differentiate to osteoclasts with high resorption activity by RANKL alone [68]. Another study of mouse models of bone loss has demonstrated that IFN*γ* promotes osteoclast formation through stimulation of antigen-dependent T cell activation and secretion of RANKL and TNF*α* by T cells [28]. Interestingly, the Th1-derived cytokine has also been implicated in orthodontic tooth movement, which induces local inflammation in the periodontium, by increasing the number of osteoclasts [71]. Besides osteoclastogenesis, increased apoptosis of osteoblasts induced by IFN*γ* and TNF*α* has been demonstrated in mouse models of periodontitis [69, 70]. These lines of evidence along with our study indicate that IFN*γ* and TNF*α* promote net bone degradation through increased bone resorption by osteoclasts and deficient bone formation by osteoblasts in inflamed periodontal tissue.

## 5. Conclusion

Here, we showed that costimulation with the inflammatory cytokines IFN*γ* and TNF*α* induced apoptosis in MC3T3-E1 osteoblasts via cytochrome *c* release from mitochondria, caspase activation, and Bcl-2 suppression. These findings advance our understanding of the molecular mechanism driving inflammatory bone loss in periodontal disease.

## Data Availability

The data used to support the findings of this study are available from the corresponding author upon request.
